# Supplementary figures and images for: Average annual costs of Rheumatoid Arthritis estimated by inverse probability weighting and their influence factors: A cross-sectional study based on Chinese Registry of Rheumatoid arthritis (CREDIT) Cohort
Source: PLoS One. 2025 Aug 25;20(8):e0330261. doi: 10.1371/journal.pone.0330261 (PMC12377572; doi:10.1371/journal.pone.0330261)

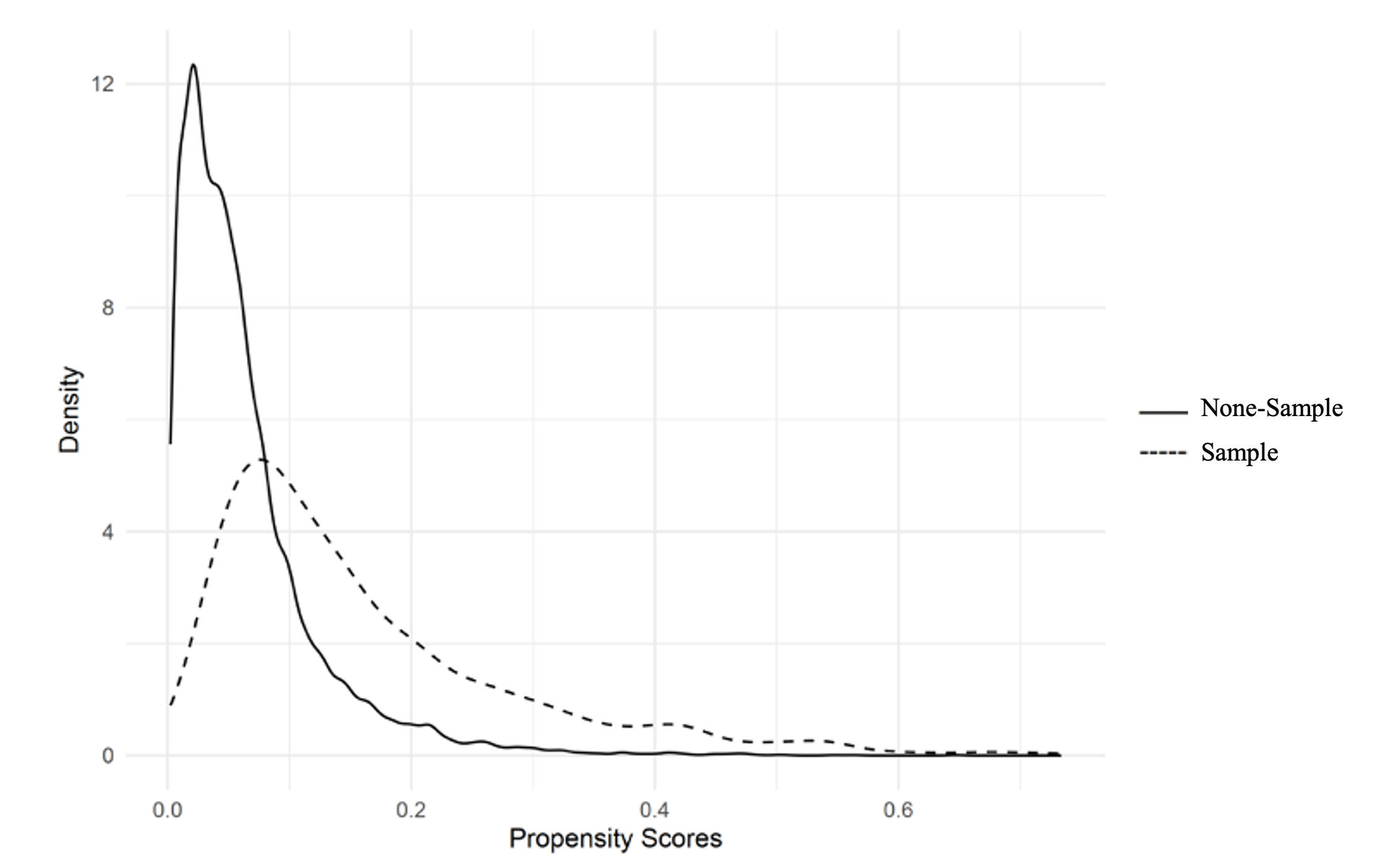

Supplement: S1 Fig — (TIF) [file pone.0330261.s001.tif]
