# Supplementary material for: Average annual costs of Rheumatoid Arthritis estimated by inverse probability weighting and their influence factors: A cross-sectional study based on Chinese Registry of Rheumatoid arthritis (CREDIT) Cohort
Source: PLoS One. 2025 Aug 25;20(8):e0330261. doi: 10.1371/journal.pone.0330261 (PMC12377572; doi:10.1371/journal.pone.0330261)
Supplement: S1 Table — (DOCX) [file pone.0330261.s002.docx]

Average Annual Costs of Rheumatoid Arthritis Estimated by Inverse Probability Weighting and Their Influence Factors: A Cross-Sectional Study Based on Chinese Registry of Rheumatoid arthritis (CREDIT) Cohort.

**S1 Tables. All the patients’ demographics and clinical characteristics and comparison between sample and none-sample, weighted sample and weighted non-sample.**

| Characteristics | RA target patients during the study period on CREDIT | Sample | None-sample | *P* Value | SMD |  | IPW | | |
| --- | --- | --- | --- | --- | --- | --- | --- | --- | --- |
|  |  |  |  |  |  |  | *weighted*  *sample* | weighted  *None-sample* | SMD |
|  | (N=18507) | (N=1293) | (N=17214) |  |  |  | （N=18465） | （N=14575） |  |
| Age, mean (SD) | 52.66(13.05) | 47.73(13.86) | 53.03(12.92) | <0.001* | 0.395^#^ |  | 52.69 (13.07) | 51.34 (13.08) | 0.104 |
| Gender, n(%) |  |  |  | 0.918 | 0.004 |  |  |  | 0.056 |
| Male | 3290(17.8) | 228(17.6) | 3062(17.8) |  |  |  | 3276 (17.7) | 2905 (19.9) |  |
| Female | 15217(82.2) | 1065(82.4) | 14152(82.2) |  |  |  | 15188 (82.3) | 11669 (80.1) |  |
| Geographical regions, n(%) |  |  |  | <0.001* | 0.211^#^ |  |  |  | 0.062 |
| Western | 4790(25.9) | 271(21.0) | 4519(26.3) |  |  |  | 4797 (26.0) | 3483 (23.9) |  |
| Central | 6022(32.5) | 359(27.8) | 5663(32.9) |  |  |  | 6009 (32.5) | 5129 (35.2) |  |
| Eastern | 7695(41.6) | 663(51.3) | 7032(40.9) |  |  |  | 7659 (41.5) | 5963 (40.9) |  |
| Medical insurance, n(%) |  |  |  | <0.001* | 0.367^#^ |  |  |  | 0.211^#^ |
| No | 6008(32.5) | 230 (17.8) | 5778 (33.6) |  |  |  | 6058 (32.8) | 3671 (23.3) |  |
| Yes | 12499(67.5) | 1063 (82.2) | 11436 (66.4) |  |  |  | 12439(67.2) | 12070 (76.7) |  |
| Duration (years), median [p25, p75] | 3.00 [0.00, 8.00] | 3.00 [1.00, 7.00] | 3.00 [0.00, 8.00] | 0.931 | 0.013 |  | 3.00 [0.00, 8.00] | 2.00 [0.00, 7.00] | 0.043 |
| Newly treated patients, n(%) | 4316(23.3) | 259(20.0) | 4057 (23.6) | 0.004* | 0.086 |  | 4345 (23.5) | 3530 (24.2) | 0.016 |
| History of comorbidity, n(%) |  |  |  |  |  |  |  |  |  |
| Fragility fracture | 231(1.2) | 20(1.5) | 211(1.2) | 0.383 | 0.027 |  | 224 ( 1.2) | 183 ( 1.3) | 0.004 |
| Joint replacement | 291(1.6) | 21(1.6) | 270(1.6) | 0.969 | 0.004 |  | 286 ( 1.5) | 206( 1.4) | 0.011 |
| Neoplasms | 191(1.0) | 19(1.5) | 172(1.0) | 0.141 | 0.043 |  | 186 ( 1.0) | 191 ( 1.3) | 0.028 |
| Allergy disease | 1089(5.9) | 99(7.7) | 990(5.8) | 0.006* | 0.076 |  | 1077 ( 5.8) | 980 ( 6.7) | 0.037 |
| Diabetes | 937(5.1) | 43(3.3) | 894(5.2) | 0.004* | 0.093 |  | 941( 5.1) | 567 ( 3.9) | 0.058 |
| Hypertension | 2884(15.6) | 156(12.1) | 2728(15.8) | <0.001* | 0.109 |  | 2887 (15.6) | 2162 (14.8) | 0.022 |
| Hyperlipidemia | 739(4.0) | 70(5.4) | 669(3.9) | 0.008* | 0.073 |  | 730 ( 4.0) | 633 ( 4.3) | 0.019 |
| Family history of RA, n(%) | 713(3.9) | 90(7.0) | 623(3.6) | <0.001* | 0.15 |  | 704 ( 3.8) | 680 ( 4.7) | 0.042 |
| DAS28-CRP, mean (SD) | 3.65(1.55) | 3.27(1.51) | 3.68(1.55) | <0.001* | 0.268^#^ |  | 3.66 (1.55) | 3.61 (1.51) | 0.032 |
| RF, median [IQR] | 92.40 [31.00, 236.00] | 91.00 [32.90, 200.00] | 93.00 [31.00, 238.00] | 0.325 | 0.024 |  | 91.96 [31.00, 236.00] | 92.05 [30.10, 235.00] | 0.031 |
| ESR, median [IQR]mm/h | 23.00 [12.00, 45.00] | 20.00 [10.00, 36.00] | 24.00 [12.00, 45.00] | <0.001* | 0.101 |  | 23.00 [12.00, 45.00] | 23.00 [12.00, 41.62] | 0.039 |
| CRP , median [IQR]mg/dl | 5.43 [1.94, 16.03] | 3.48 [1.30, 10.35] | 5.76 [2.00, 16.60] | <0.001* | 0.065 |  | 5.60 [2.00, 16.30] | 4.89 [1.75, 14.70] | 0.025 |
| Patient pain VAS score, median [IQR] | 3.80 [2.20, 5.30] | 3.20 [1.70, 5.10] | 3.80 [2.30, 5.30] | <0.001* | 0.187 |  | 3.80 [2.20, 5.30] | 3.60 [2.00, 5.20] | 0.029 |
| Global disease VAS score (patient), median [IQR] | 3.80 [2.20, 5.40] | 3.30 [2.00, 5.10] | 3.90 [2.30, 5.40] | <0.001* | 0.16 |  | 3.80 [2.20, 5.40] | 3.80 [2.00, 5.40] | 0.005 |
| Global disease VAS score (physician), median [IQR] | 3.80 [2.10, 5.40] | 3.10 [1.80, 5.10] | 3.80 [2.20, 5.40] | <0.001* | 0.182 |  | 3.80 [2.20, 5.40] | 3.60 [2.00, 5.30] | 0.045 |
| Disease activity, n(%) |  |  |  | <0.001* | 0.262^#^ |  |  |  | 0.066 |
| Remission | 5173(29.0) | 491(39.5) | 4682(28.2) |  |  |  | 5138 (28.9) | 4206 (30.5) |  |
| Low | 2544(14.3) | 180(14.5) | 2364(14.2) |  |  |  | 2541(14.3) | 1928 (14.0) |  |
| Moderate | 6761(37.9) | 405(32.6) | 6356(38.3) |  |  |  | 6757 (38.0) | 5379 (39.0) |  |
| High | 3363(18.8) | 167(13.4) | 3196(19.3) |  |  |  | 3365(18.9) | 2285 (16.6) |  |
| Treatment, n(%) |  |  |  |  |  |  |  |  |  |
| csDMARDs | 11701(63.2) | 1049(81.1) | 10652(61.9) | <0.001* | 0.436^#^ |  | 11651 (63.1) | 10264 (70.4) | 0.156 |
| NSAIDs | 2532(13.7) | 144(11.1) | 2388(13.9) | 0.007* | 0.083 |  | 2541(13.8) | 2060 (14.1) | 0.011 |
| bDMARDs/tsDMARDs | 4505(24.3) | 417(32.3) | 4088(23.7) | <0.001* | 0.190 |  | 4120(22.3) | 3588 (24.6) | 0.054 |
| Glucocorticoid | 4129(22.3) | 284(22.0) | 3845(22.3) | 0.783 | 0.009 |  | 4478(24.3) | 3732 (25.6) | 0.031 |
| Drug for osteoporosis | 3921(21.2) | 223(17.2) | 3698(21.5) | <0.001* | 0.107 |  | 3931 (21.3) | 3301 (22.6) | 0.033 |
