# Supplementary material for: Average annual costs of Rheumatoid Arthritis estimated by inverse probability weighting and their influence factors: A cross-sectional study based on Chinese Registry of Rheumatoid arthritis (CREDIT) Cohort
Source: PLoS One. 2025 Aug 25;20(8):e0330261. doi: 10.1371/journal.pone.0330261 (PMC12377572; doi:10.1371/journal.pone.0330261)
Supplement: S2 Table — (DOCX) [file pone.0330261.s003.docx]

Average Annual Costs of Rheumatoid Arthritis Estimated by Inverse Probability Weighting and Their Influence Factors: A Cross-Sectional Study Based on Chinese Registry of Rheumatoid arthritis (CREDIT) Cohort.

**S2 Table. Annual per capital costs estimated by the IPW pseudo-population RA patients (Unit: CNY)**

|  | **Mean±SD** | **Median（IQR）** | **Bootstrap Mean  (95%CI)** |
| --- | --- | --- | --- |
| **Direct costs** | **32375 ± 82197** | **12200 (5682, 32601)** | **32448(28412-37030)** |
| **Direct medical cost** | **28719 ± 78161** | **9986 (4397, 28139)** | **28792(24893-33208)** |
| Physicians' visit expense | 377 ± 939 | 120 **(**50, 300) | 377(330-428) |
| Bed expense for hospitalization | 118 ± 404 | 0 **(**0, 0) | 118(97-141) |
| Laboratory or imaging, or antibody test | 6512 ± 18173 | 1980 **(**704, 6000) | 6522(5584-7567) |
| Medication expense | 17155 ± 65862 | 5000 **(**1950, 14617) | 17188(13930-21100) |
| Other medical expense | 4557 ± 22492 | 0 **(**0, 1092) | 4587(1681-3418) |
| **Direct non-medical cost** | **3657 ± 9984** | **1200 (380, 3750)** | **3656(3130-4251)** |
| Transportation expense | 1302 ± 3329 | 392 **(**90, 1280) | 1302(1125-1499) |
| Food expenses | 998 ± 3418 | 300 **(**80, 900) | 998(822-1202) |
| Accommodation expense | 711 ± 3235 | 0 **(**0, 360) | 714(546-909) |
| Other non-medical expense | 645 ± 2793 | 0 **(**0, 0) | 642(497-806) |
| **Indirect costs** | **9520 ± 21488** | **1488 (0, 6736)** | 9523(8343-10726) |
| **Total costs** | **41895 ± 91238** | **17272 (7582, 48752)** | **41971(37107-47046)** |
