# Supplementary material for: Average annual costs of Rheumatoid Arthritis estimated by inverse probability weighting and their influence factors: A cross-sectional study based on Chinese Registry of Rheumatoid arthritis (CREDIT) Cohort
Source: PLoS One. 2025 Aug 25;20(8):e0330261. doi: 10.1371/journal.pone.0330261 (PMC12377572; doi:10.1371/journal.pone.0330261)
Supplement: S3 Table — (DOCX) [file pone.0330261.s004.docx]

Average Annual Costs of Rheumatoid Arthritis Estimated by Inverse Probability Weighting and Their Influence Factors: A Cross-Sectional Study Based on Chinese Registry of Rheumatoid arthritis (CREDIT) Cohort.

**S3 Table. Average Annual costs estimated by the IPW population of RA patients in China (Unit: CNY)**

|  | Mean (bootstrap method) | 95%CI (bootstrap method) | Proportion of direct costs(%) |
| --- | --- | --- | --- |
| Direct cost | 32448 | [28412,37030] | 60.0 |
| Direct medical cost | 28792 | [24893,33208] | 53.2 |
| Direct non-medical cost | 3656 | [3130,4251] | 6.8 |
| Indirect cost^a^ | 21649 | [19086,24552] | 40.0 |
| Total cost | 54097 | [48364,60552] | 100.0 |

^a^ estimate by average per capital Gross domestic product (GDP)
