# Supplementary material for: Average annual costs of Rheumatoid Arthritis estimated by inverse probability weighting and their influence factors: A cross-sectional study based on Chinese Registry of Rheumatoid arthritis (CREDIT) Cohort
Source: PLoS One. 2025 Aug 25;20(8):e0330261. doi: 10.1371/journal.pone.0330261 (PMC12377572; doi:10.1371/journal.pone.0330261)
Supplement: S4 Table — (DOCX) [file pone.0330261.s005.docx]

Average Annual Costs of Rheumatoid Arthritis Estimated by Inverse Probability Weighting and Their Influence Factors: A Cross-Sectional Study Based on Chinese Registry of Rheumatoid arthritis (CREDIT) Cohort.

**S4 Table. Average Annual costs estimated by the IPW (logistic regression model) population of RA patients in China (Unit: CNY)**

|  | Mean (bootstrap method) | 95%CI (bootstrap method) | Proportion of direct costs(%) |
| --- | --- | --- | --- |
| Direct cost | 34114 | [29530,39642] | 77.0 |
| Direct medical cost | 30362 | [25972,35695] | 68.5 |
| Direct non-medical cost | 3752 | [3261,4308] | 8.5 |
| Indirect cost | 10201 | [8902,11650] | 23.0 |
| Total cost | 44315 | [39113,50410] | 100 |
