# Supplementary material for: Average annual costs of Rheumatoid Arthritis estimated by inverse probability weighting and their influence factors: A cross-sectional study based on Chinese Registry of Rheumatoid arthritis (CREDIT) Cohort
Source: PLoS One. 2025 Aug 25;20(8):e0330261. doi: 10.1371/journal.pone.0330261 (PMC12377572; doi:10.1371/journal.pone.0330261)
Supplement: Attachment — Submitted filename: renamed_7ac2a.pdf [file pone.0330261.s006.pdf]

中国医学科学院基础医学研究所
北京协和医学院基础学院
Institute of Basic Medical Sciences Chinese Academy of Medical Sciences
& School of Basic Medicine Peking Union Medical College

地址：北京市东单三条5号 邮编：100005 5 Dong Dan San Tiao Beijing 100005, China Tel:8610-65296911 Fax:8610-65256546

中国医学科学院基础医学研究所
北京协和医学院基础学院
伦理审查委员会项目评审报告

项目编号： **063-2020**

经中国医学科学院基础医学研究所&北京协和医学院基础学院伦理审查委员会评审，认为下列研究项目的风险与预期利益的权衡是合理的，获取知情同意的程序符合伦理学原则。此项目通过伦理委员会审查。

项目名称： 风湿免疫疾病患者经济负担在线调查

项目负责人： 王艳红 副研究员

单 位： 中国医学科学院基础医学研究所/北京协和医学院基础学院

部 门： 流行病与卫生统计学系

合作单位： 北京协和医院

资金来源： 中国医学科学院医学与健康科技创新工程

批准日期： 2020-12-18

中国医学科学院基础医学研究所
北京协和医学院基础学院
伦理审查委员会

（印章：中国医学科学院基础医学研究所 北京协和医学院基础学院 伦理审查委员会）

2020年12月18日

中国医学科学院基础医学研究所
北京协和医学院基础学院
Institute of Basic Medical Sciences Chinese Academy of Medical Sciences
& School of Basic Medicine Peking Union Medical College

地址：北京市东单三条5号 邮编：100005 5 Dong Dan San Tiao Beijing 100005, China Tel: 8610-65296911 Fax: 8610-65256546

INSTITUTIONAL REVIEW BOARD OF INSTITUTE OF BASIC MEDICAL SCIENCES CHINESE ACADEMY OF MEDICAL SCIENCES

Project No: 063-2020

PROJECT TITLE: Economic Burden of Rheumatic Diseases
PRINCIPAL INVESTIGATOR OF PROJECT: Associate Prof. Yanhong Wang
INSTITUTE: Institute of basic medical sciences, Chinese academy of medical sciences
FUNDING AGENCY: CAMS Innovation Fund for Medical Sciences
DATE APPROVED: Dec. 18, 2020

The INSTITUTIONAL REVIEW BOARD of Institute of Basic Medical Sciences, Chinese Academy of Medical Sciences has reviewed the proposed use of human subjects in the above-mentioned project.

The IRB has approved the entirety of the project and finds it concurrent with all related protocols.

Signature: Zhao Xiaomei

Executive Chair, Institutional Review Board of IBMS, CAMS

Date: Dec. 18, 2020
